# Systemic Venous Congestion Reviewed

**DOI:** 10.7759/cureus.43716

**Published:** 2023-08-18

**Authors:** Prakash Banjade, Ashish Subedi, Shekhar Ghamande, Salim Surani, Munish Sharma

**Affiliations:** 1 Department of General Medicine, Manipal College of Medical Sciences, Pokhara, NPL; 2 Department of Internal Medicine, Gandaki Medical College, Kathmandu, NPL; 3 Department of Pulmonary, Critical Care and Sleep Medicine, Baylor Scott and White Medical Center, Temple, USA; 4 Department of Anesthesiology, Mayo Clinic, Rochester, USA; 5 Department of Medicine, Texas Agricultural and Mechanical (A&M) University, College Station, USA; 6 Department of Medicine, University of North Texas, Dallas, USA; 7 Department of Internal Medicine, Pulmonary Associates, Corpus Christi, USA; 8 Department of Clinical Medicine, University of Houston, Houston, USA; 9 Department of Pulmonary and Critical Care Medicine, Baylor Scott and White Medical Center, Temple, USA

**Keywords:** volume overload, intravascular volume, venous excess ultrasound score, point of care ultrasound, systemic venous congestion

## Abstract

Accurate determination of intravascular volume status is challenging in acutely ill patients. Favorable patient outcome is vital to correctly identify intravascular volume depletion and avoid systemic venous congestion. Most of the conventional means of hemodynamic monitoring in the acute healthcare setting are geared toward addressing the cardiac output and maintaining an optimum mean arterial pressure. While assessing and maintaining cardiac output in an acutely ill patient is very important, a venous congestion cascade is often overlooked, which can negatively affect the intraabdominal end organs. The prospect of using point-of-care ultrasound (POCUS) to determine systemic venous congestion could be a potentially handy tool for clinicians. Venous excess ultrasound score (VExUS) has also been utilized by clinicians as a semi-quantitative assessment tool to assess fluid status. This review aims to discuss the potential role of POCUS and VExUS scores in determining systemic venous congestion through a narrative review of recently published literature.

## Introduction and background

**Figure 1:**
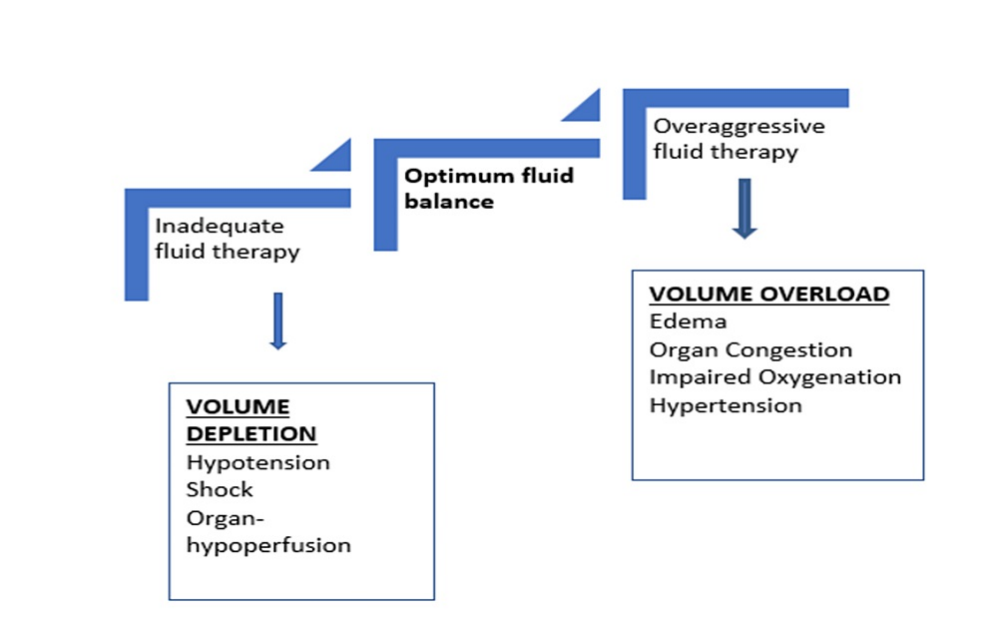
Deleterious effects of intravascular volume depletion and volume overload

The current focus in managing critically ill patients relies on maintaining mean arterial pressure, thereby addressing cardiac output and systemic vascular resistance. Clinicians have challenges in accurately assessing and managing systemic venous congestion to avoid its deleterious effects on vital organ perfusion. Optimal fluid management is vital for acutely ill patients to avoid the negative effects of both insufficient and aggressive fluid therapy (Figure 1) [1].

Some of the current tools that help measure the intravascular volume include laboratory parameters such as brain natriuretic peptide (BNP), chest imaging, pre-load assessment with inferior vena cava (IVC) diameter, and central venous pressure (CVP), among others. However, several clinical factors might influence these measures and lead to inaccurate assessment of systemic venous congestion.

With rapidly growing interest in point-of-care ultrasound (POCUS), acute care providers have been leaning towards bedside parameters of hemodynamic assessment and fluid responsiveness. Venous excess ultrasound score (VExUS) has also been utilized by clinicians as a semi-quantitative assessment tool to assess the fluid status and mainly predict acute kidney injury (AKI) in acutely ill patients due to cardiac causes. Current literature regarding POCUS parameters and systemic venous congestion assessment is still a relatively new field. We aim to revisit the concept of systemic venous congestion, mention conventionally used parameters for intravascular volume assessment, and discuss the potential role of POCUS and VExUS scores in determining systemic venous congestion.

## Review

Systemic venous congestion

Maintaining a stable cardiac output is essential for organ perfusion, achieved through the interplay of mean arterial pressure and peripheral venous resistance. The venous structure is a reservoir accommodating approximately two-thirds of blood volume at rest [2]. The clinical symptoms of venous congestion can be used as an indicator of hemodynamic changes that may predict the deterioration of renal function, the need for rehospitalization, and an increased risk of mortality after discharge in cases of acute decompensated heart failure (ADHF) [3]. One of the leading pathophysiologies of venous congestion is an increase in systemic vascular resistance, which leads to an increase in afterload. As a result, the capacity of the large veins to hold blood decreases while the amount of blood returning to the heart through the veins increases, leading to an increase in pre-load [3,4]. Another mechanism involves cardiorenal dysfunction, resulting in a rise in the overall blood volume and ultimately leading to venous congestion. Medication and dietary non-compliance also account for fluid and salt retention. It is unclear whether the elevation of neurohormonal markers in venous congestion is a causal or secondary effect [5]. During venous congestion, fluid moves from a vascular bed to extra-vascular space. Additionally, increased pressure in the veins located in the neck can hinder the flow of lymphatic fluid, leading to the potential of developing congestive heart failure [6].

Importance of understanding venous congestion

**Figure 2:**
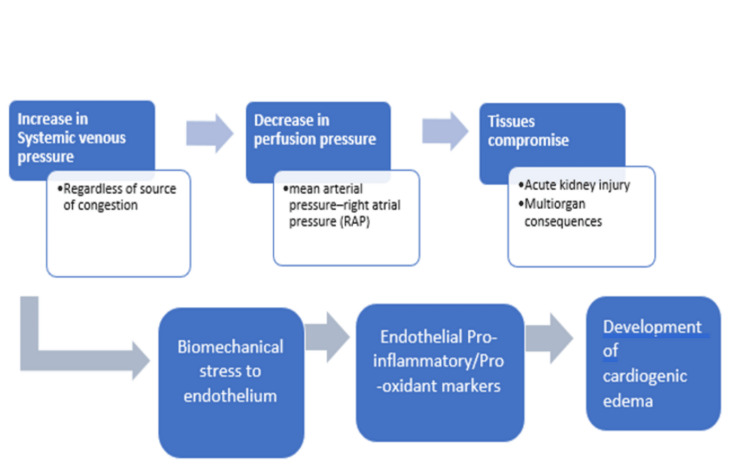
Intricate interplay between increased systemic venous pressure and organ injury

Venous congestion decreases arteriovenous gradient, ultimately affecting organ perfusion [6,7]. A dysfunctional endothelial barrier leads to a prolonged increase in capillary hydrostatic pressure. This leads to worsening interstitial edema [6-8]. Thus, an increase in systemic venous pressure causes a decrease in organ perfusion pressure resulting in a potential injury to the capsulated end organs, such as the kidney and brain [9]. Widespread endothelial damage and release of proinflammatory cytokines can trigger further tissue edema that results in impaired tissue oxygenation by diffusion [10]. Elevated CVP results in lower oxygen saturation in the brain, which is associated with poorer outcomes in post-cardiac arrest patients. Fluid overload with underlying venous congestion and cardiac dysfunction accounts for significant morbidity and mortality in ICU patients [10,11]. Studies have shown a connection between a gradual increase in fluid balance, an increase in extra-vascular lung water, and a higher mortality rate [12]. In another study, CVP-targeted aggressive fluid resuscitation reduced organ failure and improved survival [13]. During the first 72 hours of ICU stay, a positive fluid balance of 1 liter was associated with a 10% increase in mortality in a European multicenter study [13,14]. Figure 2 illustrates the effect of increased systemic venous pressure in organ perfusion.

Currently available indices for volume assessment and their limitations

Clinicians have traditionally depended on physiological parameters such as heart rate and blood pressure for volume assessment, but these parameters have been proven to be unreliable for guiding fluid replacement therapy [10,11,15). A meta-analysis [12] to assess the usefulness of a patient's history, physical examination, and diagnostic tests in identifying heart failure and volume overload showed that paroxysmal nocturnal dyspnea, orthopnea, and peripheral edema were the symptoms that were most useful among all other presenting symptoms. However, very few findings have clinical relevance in states of volume depletion caused by non-blood loss disorders, necessitating additional laboratory and diagnostic testing. Oliguria may be present in both hypovolemic and hypervolemic states. Thus, history and clinical examination are fundamental in assessing volume status, but they may not always suffice to conclude. Details are shown in Table 1.

**Table 1:** Currently utilized tools of volume assessment POCUS: point-of-care ultrasound, CRT: capillary refill time, JVP: jugular venous pressure, ABG: arterial blood gas, IVC: inferior vena cava, CVP: central venous pressure, PCWP: pulmonary capillary wedge pressure [1,15,16]

History	Presenting symptoms like diarrhea, vomiting, and bleeding, history of weight gain, history of heart failure or cardiac diseases in the past, assessing daily weights, urine output
Physical examination	Measuring blood pressure and pulse, skin and mucous membrane, CRT, JVP, precordial auscultation of third heart sounds, lung auscultation for crackles, lower extremity edema, mentation, temperature of extremities
Lab parameters	ABG analysis, plasma osmolality and plasma colloid oncotic pressure, electrolytes, urine analysis, serum, uric acid, natriuretic peptides, blood lactate
POCUS	Lung examination for congestion IVC assessment for diameter and collapsibility, internal jugular vein distension, left ventricle contractility
Pre-load evaluation	Barometric CVP, PCWP, volumetric, transpulmonary thermodilution, Swan-Ganz
Dynamic variables	Stroke volume variation, pulse pressure variation, plethysmographic variability index
Modified fluid challenge	Passive leg raises, mini fluid bolus (100-200 ml)
Imaging	Chest X-ray

Patients can present with hypotension in both hypervolemic and hypovolemic states. Interpreting postural hypotension can be challenging in elderly patients with postural dizziness due to its association with a broader range of pathologies. Above the 100 pg/ml value, BNP fails to be reliable because it lacks specificity [17]. Studies suggest that even large volumes of hemorrhage can be reliably detected by tachycardia when supine [18]. Despite the loss of 1150 ml of blood, hypotension or tachycardia are often absent [18]. Fluid replacement may not always be necessary for low blood pressure. Euvolemic patients in the emergency department showed wide variations in orthostatic measurements [19].

The degeneration of elastin with aging makes it an unreliable indicator of dehydration [20]. Other findings like dry axilla and skin mottling are not sensitive indicators of dehydration. Mild to moderate hypovolemia may not demonstrate a delayed capillary refill time [20,21]. In addition, dehydration severity cannot be co-related with patella examination [22]. Patients with hypervolemia and cardiovascular disease can benefit from assessing any elevation in JVD, although its absence doesn't rule out congestion [23]. Although the finding of raised JVD can be associated with the worst outcome in heart failure, its co-relationship with CVP is questionable and challenging among obese individuals with short necks [24]. Auscultation of third heart sound has a low negative predictive value for a pulmonary capillary wedge pressure (PCWP) >20 mm of hg [25]. In another study involving 43 patients with raised jugular venous pressure measured by PCWP, 18 patients didn't have any rales [26].

Volume-depleted states can present with high plasma osmolality, but lower osmolality can be associated with any volume state [27]. Hyponatremia also has a broader etiology [28]. Tissue hypoxia can result in type A lactic acidosis, but type B lactic acidosis is associated with a wider range of pathologies [29]. Studies have consistently failed to show the correlation of urinary markers of volume status with plasma osmolality and sodium concentration [30].

To objectively determine the patient's fluid state, physicians mostly rely on IVC and CVP measurement. Dilated IVC is not always a sign of systemic venous congestion because there are many other factors to consider. The IVC's diameter is higher physiologically in athletes. Furthermore, respiratory movements, mechanical ventilation, valvulopathies, pressure in the pulmonary circulation, and intraabdominal pressure can all affect this measurement [31]. Studies have shown that CVP has a very weak correlation with blood volume and clinical choices about fluid management should not be made with CVP [32]. Especially in critically ill patients, such frequently observed indicators do not accurately reflect their blood volume status. These measures are prone to measurement error and do not trigger the start and endpoint of fluid resuscitation [33].

Though many parameters have been used to measure patients' volume status, none accurately determine systemic venous congestion.

What is venous excess ultrasound (VExUS)?

Systemic venous pressure is an essential determinant of organ perfusion. Patients with right ventricular failure, pulmonary hypertension, and individuals with fluid overload are more likely to develop clinically severe organ congestion. Recognizing early indications of venous congestion can assist in minimizing fluid delivery, exploring underlying causes of right heart failure, and reducing the risk of significant end-organ damage, such as AKI. This is particularly important in critically ill patients whose fluid retention is exacerbated by renal impairment. The current trend among intensivists uses POCUS-based hemodynamic monitoring and fluid responsiveness assessment. VExUS protocol includes IVC diameter, hepatic vein Doppler assessment, portal vein Doppler assessment, and intrarenal vein Doppler assessment as the four components.

Origin of VExUS and scoring system

Table 2 describes the VExUS scoring system based on the severity. In mild abnormality of the hepatic vein Doppler, systolic phase amplitude is less than the diastolic phase toward the liver. In a severe abnormality, there is a reverse systolic phase. In a mild abnormality of portal vein Doppler, pulsatility fraction (PF) is 30-49%. However, in a severe abnormality, PF is >50%. In a mild abnormality of intrarenal venous Doppler, there is a discontinuous pattern in both the systolic and diastolic phases. In a severe abnormality, the discontinuous pattern is only in the diastolic phase.

**Table 2:** Mild versus severe abnormality of the hepatic, portal, and renal veins PF: pulsatility fraction

	Mild abnormality	Severe abnormality
Hepatic vein Doppler	Systolic phase amplitude < diastolic phase, toward the liver	Reverse systolic phase
Portal vein Doppler	PF 30-49%	PF>50%
Intrarenal venous Doppler	Discontinuous pattern in both systolic and diastolic phase	Discontinuous pattern in the diastolic phase

VExUS score was validated in a single-center prospective study where they found a strong association with severe flow abnormalities in multiple Doppler patterns along with dilated IVC and AKI [34]. They established a prototypical venous excess ultrasonography grading system of the degree of venous congestion to confirm its potential clinical utility in foretelling the occurrence of acute kidney damage. Data were gathered from the patient cohort at the tertiary cardiac center. Patients who had undergone heart surgery using cardiopulmonary bypass and were older than 18 years old were included in the study. Repeated POCUS examinations of the IVC, hepatic vein, portal vein, and intra-renal vein were performed. When compared to other combinations of ultrasonographic characteristics, severe flow anomalies in various Doppler patterns with dilated IVC presented the highest connection with the occurrence of ensuing AKI [34].

The flow in the hepatic vein follows a pattern similar to CVP waveforms. It has a retrograde A wave, followed by anterograde S and D waves. When a patient is healthy, the S wave should be significantly bigger than the D wave, and both waves should exhibit negative deflections as blood moves away from the probe. The magnitude of the S wave will decrease and eventually become positive in cases of severe congestion. The blood flow at this point will be away from IVC. There is a monophasic constant flow with minimal portal and renal system variation. In the case of congested portal veins, the flow becomes pulsatile; congested renal veins result in a diastolic flow [34].

The VExUS grading system prototypes include a combination of IVC diameter and venous Doppler waveform of the portal, hepatic, and interlobular renal veins. The prototype grading system was named VExUS "A" through "E," with multiple grades within each grading system (Table 3) [34].

**Table 3:** Currently used VExUS scoring system VExUS: venous excess ultrasound score, IVC: inferior vena cava, 0: no congestion, 1: mild congestion, 2: moderate congestion, 3: severe congestion [34]

Grade	VExUS A	VExUS B	VExUS C	VExUS D	VExUS E
0	IVC diameter <2cm	IVC diameter <2 cm	IVC diameter <2 cm	-	-
1	IVC ≥2 cm, normal Doppler waveforms	IVC ≥2 cm, normal Doppler waveforms	IVC ≥2 cm, normal or mild Doppler waveforms	Normal Dopplers	Normal or mild abnormality
2	IVC >2 cm, mild abnormality in at least one Doppler	IVC >2 cm, mild or severe abnormality in at least one Doppler	IVC >2 cm, severe abnormalities in one Doppler	Mild or severe abnormalities in at least one Doppler	Severe abnormalities in at least one Doppler
3	IVC >2 cm, severe abnormality in at least one pattern	IVC >2 cm, mild or severe abnormalities in multiple Doppler	IVC >2 cm, severe abnormalities in multiple Doppler	Mild or severe abnormalities in multiple Doppler	Severe abnormalities in multiple Doppler

Literature review

To find the appropriate article on the use of POCUS for detecting systemic venous congestion, we conducted a thorough search on PubMed, Google Scholar, and the Cochrane database. Our keyword search terms were "(systemic venous congestion) AND (point of care ultrasound OR venous excess ultrasound)." Table 4 shows some of the relevant studies we included for the review.

**Table 4:** Recent literature on POCUS, systemic venous congestion, and VExUS POCUS: point-of-care ultrasound, IVC: inferior vena cava, AKI: acute kidney injury, VExUS: venous excess ultrasound score, RAP: right atrial pressure, ADHF: acute decompensated heart failure, AHF: acute heart failure

Study origin and published date	Study design and author	Study population and sample size	Inferences
Canada, April 2020	Post-hoc analysis of data from a prospective cohort study, Beaubien-Souligny et al. [34]	Patients who are 18 years or older, not critically ill, and undergoing cardiopulmonary bypass, 145	By using a variety of POCUS markers, it is possible to identify venous congestion that is clinically significant. If severe flow abnormalities are present in multiple Doppler patterns along with an enlarged IVC (≥2 cm), it is considered severe venous congestion. This condition is strongly associated with the development of AKI in the future
West Indies, December 2021	Case report, Singh et al. [35]	A case of dilated cardiomyopathy with systemic venous congestion undergoing non-cardiac surgery.	VExUS identify organ congestion, which can help heart failure patients. This information guides decongestive therapy and reduces the risk of postoperative complications
Spain, February 2023	Prospective cohort, Torres-Arrese et al. [36]	AHF patients, 74	On admission, mortality can be predicted by intra-renal venous Doppler assessment, VExUS score, and pulsatility over 50%. An IVC larger than 2 cm and an intra-renal monophasic pattern can accurately predict the risk of re-admission
India, September 2020	Prospective cohort, Bhardwaj et al. [37]	Patients with cardiorenal syndrome, 30	Combined evaluation of IVC, hepatic vein, and portal vein grading could be a reliable way to display venous congestion and help guide the decision to remove the fluid
Mexico, Jan 2021	Case series, Argaiz et al. [38]	Patients with ADHF, 12	Using POCUS to assess abdominal venous congestion can assist in identifying and monitoring patients with congestive kidney injury and guide decongestive therapy in patients with ADHF
United States, May 2023	Prospective observational study, Longino et al. [39]	Patients undergoing right heart catheterization, 56	A significant connection is present between VExUS and RAP across a range of patients, providing evidence for further exploration of VExUS to evaluate venous congestion and direct treatment for various critical conditions

Beaubien-Souligny et al. [34] conducted a post-hoc analysis of data collected during a prospective cohort study at a tertiary cardiac surgery center from August 2016 to July 2017. Their investigation aimed to evaluate the effectiveness of various venous congestion grading systems, which utilized ultrasound markers, in predicting the likelihood of AKI following cardiac surgery. Patients who were 18 years or older and had undergone cardiac surgery with the use of cardiopulmonary bypass but were not in critical condition were eligible to participate. Patients who had a critical illness, AKI, or delirium prior to surgery were not included in the study. Additionally, patients with conditions that could have affected portal Doppler assessment (such as cirrhosis or portal thrombosis), as well as those with severe chronic kidney disease (with an estimated glomerular filtration rate of less than 15 mL/min using the modified diet in renal disease formula) or who were on dialysis, were also excluded. Each patient received multiple POCUS assessments. These assessments were conducted the day before surgery, upon admission to the ICU after surgery, and daily from postoperative days 1 to 3. During every ultrasound assessment, the hepatic vein Doppler, portal vein Doppler, intra-renal venous Doppler, and IVC ultrasound were conducted. They calculated the VExUS score for all of the patients. After surgery, if there was severe congestion (Grade 3), it was linked to the likelihood of developing AKI in all VExUS grading systems. It is important to note that the findings of this study may not be applicable to a wider population due to its limited scope. The study only included patients who underwent cardiac surgery at a single center. Additionally, since the analysis relied on retrospective data, certain ultrasound features such as respiratory collapsibility, three-dimensional IVC measurements, and extra-vascular lung water assessment were not incorporated into the VExUS grading systems.

A case study by Singh et al. [35] describes a 49-year-old man with a sizeable inguinoscrotal hernia that had become incarcerated. The patient was diagnosed with dilated cardiomyopathy and had an ejection fraction of 20%. Despite his condition, he was clinically stable and treated with oral furosemide, carvedilol, and enalapril. When he was admitted to the hospital, he was evaluated by medical professionals and determined to be dehydrated. As a result, his diuretics were temporarily discontinued. During the preoperative assessment conducted by the anesthesiologist, a POCUS was performed, which revealed global dilatation of both the right and left ventricles with weak ventricular contractions. Additionally, the diameter of his IVC was observed to be greater than 2.1 cm, and the collapsibility was less than 50%, indicating systemic venous congestion. The patient experiences difficulty breathing when lying flat in bed. Although they attempted to treat the hernia with noninvasive methods, the patient was administered intravenous furosemide for four consecutive days to aid in diuresis. Following the failure of conservative treatment, surgery was deemed necessary for the patient. In the operating room, the anesthesiologist utilized a handheld device to conduct another POCUS. After the surgery, a furosemide infusion of 20 mg/hr was given for four hours due to the preoperative VExUS assessment of portal vein pulsatility. As a result, the patient experienced a significant relief of dyspnea and a large diuresis of around 4500 mL over 12 hours. The VExUS was performed again after 12 hours. The results revealed a portal vein without pulsation or phasic movement, a hepatic vein with decreased regurgitant flow, and an IVC that is now collapsible, indicating some relief of venous congestion. After two days in ICU, the patient took regular diuretics and was discharged after seven days. VExUS scan played a significant role in deciding interventions for systemic venous congestion. This case highlights how an anesthesiologist could utilize VExUS during the perioperative phase to direct therapeutic measures for patients with advanced heart disease.

Torres-Arrese et al. [36] conducted a study across multiple centers to assess how helpful systemic venous ultrasonography is in predicting outcomes for patients admitted to the hospital for acute heart failure (AHF). They included patients with AHF and an NT-proBNP level above 500 pg/mL as their primary hospital diagnosis. Ultrasound scans were conducted on multiple organs within 24 hours of admission and again on discharge. Follow-up scans were also performed within the first 15 days after discharge. The primary aim of this research was to outline various ultrasound measurements and ratings, including the VExUS systemic congestion score, in patients diagnosed with AHF. The study also seeks to determine if these measurements can forecast potential complications, such as re-admissions or death, related to heart failure or otherwise. The average age of the patients was 79.5 years. Most of them, 78.4%, had an underlying cardiovascular disease, and 43.2% had a previous pulmonary disease. Upon admission, most patients were classified as NYHA III (62%), but they improved to NYHA II (37.8%) upon discharge and NYHA I (37.8%) during their follow-up clinic visits. NT-proBNP levels were measured at admission (mean 10278.5 pg/L, SD 12740), discharge (mean 6156 pg/L, SD 7889), and follow-up (mean 5438 pg/L, SD 5712). Creatinine levels were also measured at admission (1.58 mg/dL, SD 0.8), discharge (1.47 mg/dL, SD 0.94), and follow-up (1.52).

Upon admission, during discharge, and for 90 days of follow-up, ultrasounds were conducted to assess multiple organs, including the lungs, IVC, and various veins such as hepatic, portal, intra-renal, and femoral, using pulsed-wave Doppler technology. The experts used the VExUS to determine a score for systemic congestion based on the dilatation of the IVC and the pulsed-wave Doppler morphology of the hepatic, portal, and intra-renal veins. The results of specific tests, such as the intra-renal monophasic pattern, as well as a portal pulsatility greater than 50% and a VExUS score of 3 indicating severe congestion, can predict the likelihood of death during hospitalization. During a follow-up visit, it was found that an IVC measurement above 2 cm and a monophasic pattern in the kidney were strong indicators of potential re-admission related to AHF. Based on the information provided, it can be concluded that having a VExUS score of 3 upon admission may indicate a higher likelihood of death during admission, death related to heart failure, and early re-admission. However, this finding is similar to other ultrasound evaluations that are easier to perform. As this is a pilot study, it is imperative to note that only four expert sonographers were chosen to conduct the examination, which may limit the reproducibility of the findings. Nevertheless, it is clear that ultrasound imaging is an essential tool for predicting mortality and hospital re-admission in AHF patients by detecting systemic venous congestion.

Bhardwaj et al. [37] conducted a prospective study at the medical ICU to assess venous congestion using POCUS. The study only included patients over 18 years old admitted with a provisional diagnosis of cardiorenal syndrome. The study excluded patients with cirrhosis history with portal hypertension or IVC thrombus. During the study, patients received ultrasound examinations to monitor their AKI status. An experienced intensivist with at least three years of ultrasound examination experience conducted these examinations. Ultrasound assessments were performed on the IVC, hepatic vein, and portal vein at the patient's bedside until AKI was resolved or dialysis was initiated. The main goal was to evaluate how the VExUS score changed over time and its relationship with AKI in individuals with cardiorenal syndrome. The secondary objective was to evaluate the relationship between the VExUS score and fluid balance, right heart function, and clinical indications of fluid overload. The study analyzed 30 patients with AKI, whose average age was 59.53 ± 16.47. Of these patients, 46.7% were in AKI stage 1, while 26.7% were in AKI stage 2 and stage 3. The majority of patients (66.7%) had a VExUS Grade 3. Improvement in the VExUS grade was significantly linked with the resolution of AKI. Likewise, there was a significant correlation between fluid balance and changes in the VExUS grade. It was observed that in patients who experienced AKI resolution, their CVP did not return to normal. This indicates that the CVP trend may not accurately represent the patient's clinical condition due to limitations. Additionally, different patients may have varying tolerance levels for CVP, making it an unreliable indicator. In contrast, Doppler flow markers may better assess the severity of organ congestion and potential dysfunction. Based on the results above, it appears that a combined evaluation of IVC, hepatic vein, and portal vein grading could be a reliable way to display venous congestion and help guide the decision to remove the fluid. It is crucial to perform a bedside sonographic assessment of dynamic blood flow parameters, and this area of study could benefit from further interventional trials.

Twelve patients with ADHF were included in a case series by Argaiz et al. [38]. Eligible patients were those who were 18 years or older and arrived at a single tertiary care center's emergency department with a diagnosis of ADHF. This study aimed to assess changes in portal vein flow in patients with ADHF upon arrival and after receiving decongestive treatment. To evaluate systemic venous congestion, POCUS was utilized. Upon arrival, all patients underwent an initial POCUS examination which included evaluation of IVC size, collapsibility index, portal vein, and hepatic vein Doppler. The treating clinicians made the decision to use diuretic treatment based on the POCUS results. The examination was conducted again for all patients after receiving decongestive treatment. Patients were considered successfully decongested if their symptoms and signs of congestion showed clinical improvement and/or their BNP levels were lower. Six of the 12 patients had left-sided heart failure, including heart failure with reduced ejection fraction and heart failure with preserved ejection fraction. The remaining six patients had right-sided heart failure. Following the diuretic treatment, all patients showed clinical improvement and were successfully decongested. A negative cumulative fluid balance and reduced BNP levels supported this. According to this research, using POCUS to assess abdominal venous congestion can assist in identifying and monitoring patients with congestive kidney injury. If a patient presents with a full IVC and high portal vein PF alongside AKI, medical professionals should be aware of the possibility of venous congestion leading to end-organ damage.

A group of patients who underwent outpatient and inpatient right heart catheterization were examined with VExUS right before the catheterization procedure [39]. Internal medicine and emergency medicine residents who received institutional ultrasound training performed ultrasonography at the bedside. Researchers have found a strong correlation between right atrial pressure (RAP) and VExUS grade, with a statistically significant positive association. In predicting a RAP of 12 mmHg or higher, VExUS demonstrated a highly accurate area under the curve (AUC) of 0.99 (95% CI 0.96-1), compared to an AUC of 0.79 (95% CI 0.65-0.92) for IVC diameter. These findings indicate a significant connection between VExUS and RAP across a range of patients, providing evidence for further exploration and use of VExUS to evaluate venous congestion and direct treatment for various critical conditions. As elevated venous pressures are known to have negative consequences and longer ICU stays, a noninvasive, easier-to-perform method for estimating this parameter could be a useful tool for clinicians in the hospital.

Future research directions

Studies have shown that POCUS may be useful in assessing systemic venous congestion, but further research is needed to fully explore its potential. To improve early recognition and intervention, we recommend investigating the sensitivity and specificity of POCUS in detecting subtle changes that indicate early systemic venous congestion. These changes include variations in CVP, hepatic vein flow patterns, and renal venous Doppler indices. By conducting such research, we can potentially prevent disease progression and reduce morbidity and mortality. Incorporating POCUS with artificial intelligence algorithms could potentially enhance diagnostic precision, simplify workflow, and facilitate remote monitoring of patients suffering from systemic venous congestion, particularly in areas with limited resources. One way to track the progression or resolution of systemic venous congestion over time is through serial POCUS examinations. Conducting longitudinal studies can provide valuable information about the natural history of the condition, as well as how patients respond to treatment and potential indicators of clinical deterioration.

## Conclusions

Identifying the volume status of critically ill patients through systemic venous congestion is crucial, yet this approach is unfortunately underutilized. POCUS is a useful and versatile tool for evaluating systemic venous congestion. It provides non-invasive, real-time imaging that helps clinicians make prompt and accurate decisions. The evidence presented in this review supports the use of POCUS in the early detection of systemic venous congestion. Clinicians can use POCUS to identify signs of venous congestion before they become clinically apparent by evaluating parameters such as CVP, hepatic vein flow patterns, and renal venous Doppler indices. Early recognition of conditions is crucial to prevent disease progression and complications. POCUS has shown promise in evaluating systemic venous congestion, but additional research is imperative. Prospective studies must investigate the impact of POCUS on patient outcomes, and there must be an effort to standardize POCUS training and establish best practices.
